# BRAF inhibitors: resistance and the promise of combination treatments for melanoma

**DOI:** 10.18632/oncotarget.19836

**Published:** 2017-08-03

**Authors:** Merope Griffin, Daniele Scotto, Debra H. Josephs, Silvia Mele, Silvia Crescioli, Heather J. Bax, Giulia Pellizzari, Matthew D. Wynne, Mano Nakamura, Ricarda M. Hoffmann, Kristina M. Ilieva, Anthony Cheung, James F. Spicer, Sophie Papa, Katie E. Lacy, Sophia N. Karagiannis

**Affiliations:** ^1^ St John's Institute of Dermatology, Genetics and Molecular Medicine, King's College London, Guy's Hospital, Tower Wing, London, UK; ^2^ Research Oncology, School of Cancer Sciences, King's College London, Guy's Hospital, Bermondsey Wing, London, UK; ^3^ Breast Cancer Now Unit, School of Cancer Sciences, King's College London, Guy's Cancer Centre, London, UK

**Keywords:** melanoma, BRAF, MAPK, immunotherapy, CTLA-4

## Abstract

Identification of mutations in the gene encoding the serine/threonine-protein kinase, BRAF, and constitutive activation of the mitogen-activated protein kinase (MAPK) pathway in around 50% of malignant melanomas have led to the development and regulatory approval of targeted pathway inhibitor drugs. A proportion of patients are intrinsically resistant to BRAF inhibitors, and most patients who initially respond, acquire resistance within months. In this review, we discuss pathway inhibitors and their mechanisms of resistance, and we focus on numerous efforts to improve clinical benefits through combining agents with disparate modes of action, including combinations with checkpoint inhibitor antibodies. We discuss the merits of combination strategies based on enhancing immune responses or overcoming tumor-associated immune escape mechanisms. Emerging insights into mechanisms of action, resistance pathways and their impact on host-tumor relationships will inform the design of optimal combinations therapies to improve outcomes for patients who currently do not benefit from recent treatment breakthroughs.

## BRAF INHIBITORS AND RESISTANCE MECHANISMS IN PATIENTS WITH MELANOMA

Malignant melanoma is the fifth most common malignancy in the UK, making up 4% of all cancer diagnoses, with only 20% of people diagnosed with metastatic disease surviving beyond five years [1, 2]. The clinical management of patients with unresectable or metastatic melanoma has been transformed in recent years with the emergence of novel targeted and immunomodulatory therapeutics, including checkpoint blockade antibodies designed to trigger T cell activation and promote anti- tumor immune responses (Table 1). Prior to this, dacarbazine chemotherapy or combination regimens, in some cases with IL-2 or IFNα2b immunotherapy, were the only approved treatments, demonstrating short median overall survival benefits of less than 9 months [3].

**Table 1:** U.S. FDA-approved agents and combinations for the treatment of malignant melanoma

Agent/Combination (brand names)	Year of first regulatory approval	Specificity	Class	Mechanisms of action	Indication
**Chemotherapies**					
Dacarbazine(DTIC-Dome^®^)	1975	Non-specific	Alkylating agent	Interferes with cancer cells leading to DNA damage, cell cycle arrest and tumor cell apoptosis	Stage IV melanoma
**Immunotherapies**					
IFNα2b(INTRON^®^ A)	1995	IFNα Receptor 1 and 2	Cytokine	Multifunctioning immunoactivatory cytokine enhances anti-tumoral immune response, anti-angiogenic, anti-proliferative and pro-apoptotic properties	Adjuvant setting after surgery to Stage III patients free of disease, at high risk recurrence, orStage IIB or Stage IIC patients with lesions of > 4 mm Breslow thickness)
High dose IL-2 (Aldesleukin, Proleukin^®^)	1998	IL-2 receptor expressed on lymphocytes	Cytokine	Immune activating, increases activation and proliferation of immune cells (e.g. T, NK, B cells)	Advanced metastatic melanoma
Pegylated IFNα2b (Sylatron^®^)	2011	IFNα receptor 1 and 2	Cytokine	Modified (pegylated) form of IFNα2b with increased half-life and enhanced therapeutic efficacy	Microscopic or macroscopic nodal melanoma following surgical resection, including therapeutic lymph node dissection
Ipilimumab (Yervoy^®^)	2011	CTLA-4 expressed on T cells	Humanized monoclonal antibody (mAb)	Inhibition of checkpoint receptor CTLA-4 preventing engagement with CD80/CD86, activates immune system enhancing T cell activation and targeting CTLA-4-expressing Tregs	Stage III or Stage IV melanoma
Pembrolizumab (KEYTRUDA®)	2014	PD1 expressed on T cells	Humanized monoclonal antibody (mAb)	Inhibition of checkpoint receptor PD-1, prevents interaction between PD-1 and its ligands PD-L1 and PD-L2, releasing PD-1 pathway-mediated inhibition, prevents T cell anergy or deletion, activates immune system enhancing T cell activation	Unresectable Stage III melanoma or Stage IV melanoma
Nivolumab (OPDIVO®)	2014	PD1 expressed on T cells	Humanizedmonoclonal antibody (mAb)	Targets the inhibitory receptor PD-1 prevents interaction between PD-1 and its ligands PD-L1 and PD-L2, releasing PD-1 pathway-mediated inhibition, prevents T cell anergy or deletion, activates immune system enhancing T cell activation	Unresectable Stage III melanoma or Stage IV melanoma
Talimogene laharparepvec (IMLYGIC® or T-Vec)	2015	Modified oncolytic herpes virus	Targeted Oncolytic virus immunotherapy	Virus construct designed to replicate within cancer cells and produce granulocyte-macrophage colony-stimulating factor (GM-CSF) causing cell lysis and death, and releasing tumor-associated antigens. Alongside GM-CSF, this may promote an anti-tumor immune response	Local treatment of unresectable cutaneous, subcutaneous, and nodal lesions in patients with recurrent melanoma after surgery
Ipilimumab (Yervoy^®^)&Nivolumab (OPDIVO®)*(Combination)*	2015	CTLA-4 expressed on T cells & PD1 expressed on T cells		Immune checkpoint inhibitors that target separate, distinct checkpoint pathways. Inhibition of these immune checkpoint pathways results in enhanced T cell function greater than the effects of either antibody alone	Unresectable Stage III melanoma or Stage IV melanoma
**Targeted therapies of BRAF/MEK**					
Verumafenib (Zelboraf^®^)	2011	BRAF V600E, mutated form of BRAF protein	Small molecule kinase inhibitor	Blocks activity of the V600E-mutated form of BRAF, and thus the mitogen-activated protein kinase pathway, reducing proliferation of melanoma cells carrying the mutation	Unresectable Stage III melanoma or Stage IV melanoma that carry theBRAF V600E mutation
Dabrafenib(Tafinlar*)	2013	BRAF V600E mutated form of BRAF protein	Small molecule kinase inhibitor	Blocks mitogen-activated protein kinase pathway reducing proliferation of melanoma cells carrying mutation	Unresectable Stage III melanoma or Stage IV melanoma that carry theBRAF V600E mutation
Trametinib (Mekinist*)	2013	Mitogen-activated extracellular signal regulated kinase 1 (MEK1) and MEK2	Small molecule kinase inhibitor	Selective, allosteric inhibitor of mitogen-activated extracellular signal regulated kinase 1 (MEK1) and MEK2 activation and kinase activity. This extracellular signal related kinase (ERK) pathway is often activated by mutated forms of BRAF in melanoma and other cancers. Blocks mitogen-activated protein kinase pathway reducing proliferation of melanoma cells carrying mutation.	Unresectable Stage III melanoma or Stage IV melanoma that carry theBRAF V600E mutation
Dabrafenib(Tafinlar*)&Trametinib (Mekinist*)*(Combination)*	2014	BRAF V600E mutated form of BRAF protein&Mitogen-activated extracellular signal regulated kinase 1 (MEK1) and MEK2	Small molecule kinase inhibitors	Simultaneous inhibition of mutant BRAF (dabrafenib) and MEK kinases (trametinib)	Unresectable Stage III melanoma or Stage IV melanoma that carry theBRAF V600E mutation
Verumafenib (Zelboraf^®^)&Cobimetinib (COTELLIC)*(Combination)*	2015	BRAF V600E mutated form of BRAF protein&Mitogen-activated extracellular signal regulated kinase 1 (MEK1) and MEK2	Small molecule kinase inhibitors	Simultaneous inhibition of mutant BRAF (dabrafenib) and MEK kinases (cobimetinib)	Unresectable Stage III melanoma or Stage IV melanoma that carry theBRAF V600E mutation

mAb: monoclonal antibody; IFN: interferon; IL: interleukin.

**Figure 1:**
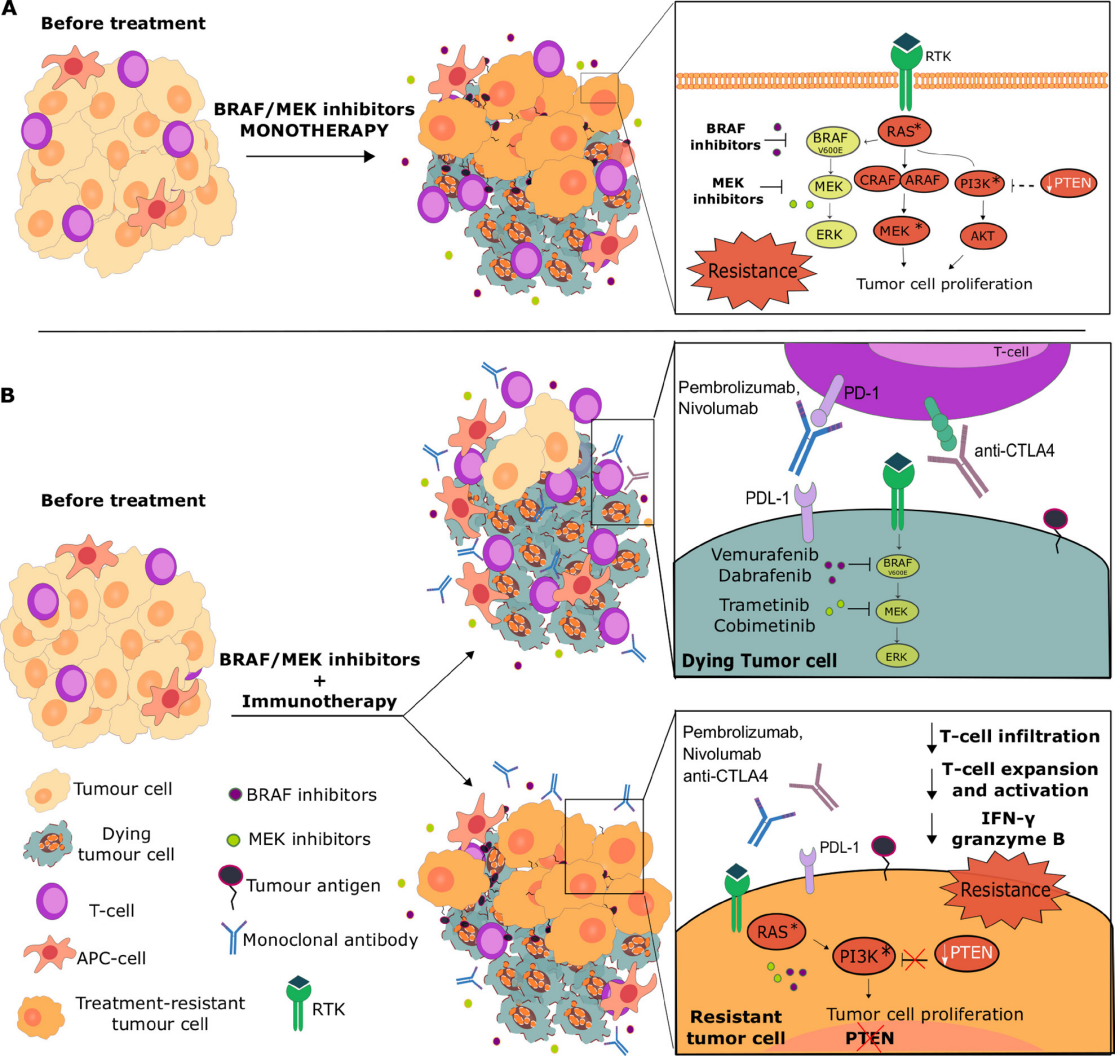
Influence of pathway resistance mechanisms on BRAF/MEK inhibitor monotherapies and combinations with immunotherapies (**A**) MAPK signaling inhibition may either result in the survival of tumor cells with activated pathways such as the PI3K/AKT, or exert strong selective pressure on melanoma tumors to acquire gain-of-function mutations, methylation or high copy number loss of tumor suppressor genes such as PTEN. These can lead to increased activity of alternative pathways (e.g. PI3K/AKT), which could confer survival advantages for BRAF/MEK inhibitor-resistant melanomas. (**B**) The success of combinations of BRAF/MEK inhibitors with immunotherapies may also depend on activation of alternative pathways to MAPK. MAPK pathway inhibitors may potentiate an anti-tumor immune response by destroying cancer cells and the tumor microenvironment, reducing tumor-associated immunosuppressive effects, enhancing IFNγ production, T cell proliferation and MHC expression, all of which could result in tumor antigen presentation and more effective anti-tumor immune responses. All or some of these could then be further enhanced with T cell activation and Treg destruction, engendered by immune checkpoint inhibition combination or subsequent treatment (top). However, in tumors with mutations on pathway genes, including those with loss of PTEN, that support alternative activation of pathways such as the PI3K/AKT, both BRAF/MEK and checkpoint blockade inhibition as monotherapies or combinations would suffer from resistance. This may be due to impaired T cell infiltration into tumors, reduced T cell activation and expansion and loss of immuno-activatory signals (e.g. reduced IFNγ, granzyme B release). These may point to shared mechanisms of resistance for BRAF inhibitors and immunotherapies and could partly explain non-responders to combinatory or sequential BRAF/MEK inhibitor and immunotherapy treatments.

Approximately 50% of melanomas carry mutations in the gene encoding BRAF, part of the MAPK pathway involved in regulating cell growth and proliferation (Figure 1A) [4]. This pathway involves a signaling cascade initiated by the binding of growth factors or cytokines to their respective receptors, resulting in activation of Ras, which then recruits Raf proteins, a family of protein kinases including BRAF, to the cell membrane [5]. Phosphorylation of Raf allows the activation of MEK1 (MAP kinase/ERK kinase 1), which positively regulates the extracellular signal-regulated kinases (ERK). ERK can then directly phosphorylate downstream transcription factors, leading to increased transcription and eventual cell growth [5].

The V600E missense valine to glutamic acid mutation accounts for approximately 80–90% of BRAF mutations [4, 6–7]. This mutation leads to a conformational change, resulting in constitutive activation of BRAF, and consequently of the MAPK/ERK pathway, promoting survival and proliferation of melanoma cells. Other BRAF mutations include V600K, V600R and V600M, estimated as being present in 7.8%, 1% and < 1% of melanomas, respectively [8, 9–10]. Other gene mutations in melanoma include NRAS, GNAQ and KIT, estimated to be present in 13–25%, 1.3% (but much more in uveal melanoma) and 2–8% (but more in acral/mucosal subtypes) of melanomas respectively [11, 12–13].

Following the discovery of the V600E mutation, targeted therapies such as the mutant BRAF-specific small molecule inhibitors vemurafenib and dabrafenib, were developed. Vemurafenib was the first targeted drug to show a survival benefit in metastatic melanoma, in the context of a phase III trial. In the phase III BRIM3 registration trial in 2011, vemurafenib was compared with dacarbazine for the first line treatment of BRAF^V600E^ mutant metastatic melanoma. The objective response rate (ORR) for vemurafenib was 48% (95% Confidence Interval [CI] 42–55) compared to 5% (95% CI, 3–9) for dacarbazine (*P* < 0.001), and the median progression-free survival (PFS) was 5.3 months vs. 1.6 months, respectively (Hazard Ratio [HR] 0.26; 95% CI 0.20–0.33) [14]. The relative risk reduction for death or disease progression was 74%, for vemurafenib compared to dacarbazine [14].

Another selective BRAF inhibitor, dabrafenib, was subsequently developed and showed similar clinical benefits. In the phase III trial of first line dabrafenib vs. dacarbazine in mutation-positive metastatic melanoma, median PFS was 5.1 months for dabrafenib, vs. 2.7 months for dacarbazine (HR 0.30; 95% CI 0.18–0.51; *P* < 0.0001). In addition, ORR was 50% vs. 3% [15].

Following on from the success of BRAF inhibitors, MEK inhibitors were subsequently developed. The first of these, trametinib, demonstrated an ORR of 22% vs. 8% for dacarbazine, and a median PFS of 4.8 months vs. 1.5 months (HR 0.45; 95% CI, 0.33–0.63; *P* < 0.001) in BRAF-mutant metastatic melanoma in the phase III METRIC trial [16]. Furthermore, cobimetinib, a selective MEK1/2 inhibitor, has been shown to act on non-phosphorylated as well as phosphorylated MEK and can reduce ERK activation. Cobimetinib was safe and demonstrated some partial responses in a Phase I trial [17]. Further development was undertaken in combination trials.

In addition to treating disseminated metastatic disease, BRAF/MEK inhibitors may also provide an effective neoadjuvant strategy for local or regional BRAF^V600E^ mutant melanoma, allowing surgical removal of previously inoperable melanomas [18]. Kolar et al. report the use of neoadjuvant vemurafenib in a patient with an initially inoperable solitary melanoma brain metastasis. Vemurafenib treatment caused substantial tumor shrinkage, allowing subsequent complete resection of the metastasis [19]. Similarly, vemurafenib therapy induced tumor regression in a patient with unresectable lymph node metastases, after which the patient became eligible for radical surgery [20]. Furthermore, dual BRAF/MEK inhibition (dabrafenib and trametinib) was successfully employed as neoadjuvant treatment for advanced ‘in transit’ melanoma [21]. However, prospective studies are required to determine whether neoadjuvant BRAF/MEK inhibitor therapy will have an impact on patient survival.

Despite improvements in progression-free survival, most patients with BRAF-mutant metastatic melanoma still demonstrated disease progression within months following treatment with BRAF or MEK inhibitor monotherapy due to development of resistance [22]. Subsequently, research aimed at understanding the ways by which tumors become resistant to BRAF^V600E^-targeted therapy has prompted the use of multiple drugs in concert to attempt to maximize survival.

### Intrinsic resistance to BRAF inhibitors

Early trials indicated that around 20% of patients with *BRAF*^V600E^ mutant melanomas did not respond to BRAF inhibitors [23]. Such patients were described as having intrinsic resistance to BRAF inhibition. Malignant melanomas are extremely heterogeneous, and tumors acquire new mutations as they move from primary lesions to metastases [24]. As a result, while some melanoma cells may carry the V600E BRAF mutation, others, within the same patient, may carry different mutations not susceptible to BRAF inhibition. Major mechanisms of intrinsic resistance include (Table 2).

**Table 2:** Proposed causes of intrinsic and acquired resistance to BRAF inhibitors

Gene mutation/cause of resistance	Proposed mechanisms of resistance
**Intrinsic mechanisms**	
Loss of PTEN	PTEN is a crucial suppressor of the PI3K/AKT pathway. Loss of PTEN leads to constitutive activation of the pathway and allows cell proliferation to continue even in the presence of BRAF inhibition.
RAC1^P29S^	RAC1^P29S^ mutation sustains MAPK signaling even in the presence of BRAF inhibitors, so cell proliferation can continue despite inhibition.
Overexpression of MAP3K8	MAP3K8 encodes the COT protein. COT can independently activate the MAPK/ERK pathway, and so increased levels of COT mean cellular proliferation continues despite BRAF inhibition.
Hepatocyte growth factor (HGF) secretion by stromal cells	Secretion of HGF by stromal cells leads to activation of MET, a receptor for HGF, reactivate the MAPK/ERK and PI3K/AKT pathways, leading to BRAF inhibitor resistance.
Loss of NF1 tumor suppressor gene	NF1 is a negative regulator of RAS signaling. Loss of NF1 by mutation allows RAS increase, subsequent CRAF activation, leading to activation of the MAPK pathway, even in the presence of BRAF inhibition.
Amplification of CCND1	CCND1 encodes Cyclin D1, a key cell cycle regulator, which can help bypass proliferation inhibition by BRAF inhibitors.
**Acquired mechanisms**	
Relief of ERK negative feedback	BRAF inhibitor arrest tumor growth by inhibiting the ERK pathway. This relieves ERK negative feedback on RAS, partially restoring RAS activity leading to RAS-induced BRAF^V600E^dimers. BRAF inhibitors bind one and transactivate the other BRAF, reducing BRAF inhibitor therapy.
RAS-activating mutations	Mutated RAS-GTP becomes constitutively active, enhances BRAF^V600E^dimerisation, reactivates the ERK pathway and confers resistance to BRAF inhibitors which only block monomeric BRAF^V600E^.
BRAF^V600E^alternative splicing	A BRAF^V600E^ splice variant p61BRAF^V600E^ due to mutations or epigenetic changes could form dimers in a RAS-independent manner, making the BRAF inhibitor ineffective as it blocks monomeric BRAF^V600E^.
BRAF^V600E^overexpression	Increased BRAF^V600E^ levels due to gene copy number gain may also promote spontaneous BRAF^V600E^dimerization, reactivating the ERK pathway and causing treatment failure in some patients.
Alternative RAF isoforms	BRAF^V600E^ melanoma treated with BRAF inhibitors may acquire resistance through flexible switching between different RAF isoforms capable of reactivating the ERK pathway, upregulating ARAF or CRAF.
COT overexpression	COT, possibly due to gene amplification or yet unidentified mechanisms, can reactivate MEK in the presence of BRAF inhibition, stimulating ERK signaling and driving resistance.
MEK-activating mutations	Activating mutations in MEK1/ MEK2 render BRAF blockage ineffective, as MEK reactivation means the MAPK/ERK pathway can still proceed downstream of BRAF regardless of its inhibition.
Adaptive PI3K/AKT signaling	Abnormal PI3K/AKT signaling is a common feature of melanomas. Blockage of ERK signaling may lead to adaptive PI3K/AKT hyperactivity that compensates for BRAF inhibition and drives resistance.
Upregulation of RTKs	The PI3K/AKT-pathway is activated by growth factors that bind to RTKs, such as PDGFR-β and IGF-1R. With BRAF blockade, tumor cells may upregulate these leading to persistent PI3K/AKT signaling.
PI3K/AKT-activating mutations	PI3K and AKT-activating mutations enhance AKT-signaling, which increases anti-apoptotic signals and upregulates expression of key proliferative genes, allowing survival signals independently of BRAF.
Enhanced EGFR signaling	Upregulation/activation of EGFR driven by suppression of SOX10 and enhanced TGF-β signaling, conferring oncogene-induced senescence, reversed with BRAF/MEK inhibition.

### Loss of PTEN

One of the most common mutations leading to intrinsic BRAF resistance is loss of the phosphatase and tensin homolog (*PTEN*) gene, which occurs in up to 35% of melanomas [25]. PTEN is a tumor suppressor and a crucial negative regulator of Phosphatidylinositol-4,5-bisphosphate 3-kinase (PI3K) [26]. Therefore, decreased responses to BRAF inhibition seen in patients with concurrent BRAF activation and PTEN loss, is thought to be due to constitutive activation of the PI3K/AKT (Protein kinase B) pathway (Figure 1A). Increased activation of this pathway leads to cellular proliferation, growth and survival [27].

### RAC1P29S mutations

RAC1 regulates cellular motility and proliferation, and is a GTPase effector of RAS. It has been suggested that the RAC1^P29S^ somatic mutation might confer BRAF resistance by sustaining MAPK signaling in the presence of BRAF inhibitors. Indeed a study by Watson et al found that a RAC1^P29S^ mutation in melanoma cell lines led to resistance to BRAF inhibition *in vitro* and *in vivo* [28]. This was confirmed in the patient setting by Van Allen et al. who found that more than a fifth of melanoma patients exhibiting intrinsic BRAF resistance, expressed mutations in RAC1 [29].

### Loss of NF1 tumor suppressor gene

NF1 is a negative regulator of RAS, the first signaling protein in the MAPK pathway. Loss of NF1 via mutation means negative inhibition of RAS stops, and RAS levels increase. This activates the protein kinase CRAF and leads to activation of the MAPK pathway [30], even in the presence of BRAF inhibition.

### Amplification of CCND1

CCND1 encodes Cyclin D1, a key protein in the regulation of the cell cycle. Cell lines with basal levels of Cyclin D1 have been reported to be less dependent on the BRAF signaling pathway [31]. Therefore, in cells with amplified CCND1 where more Cyclin D1 is produced, administration of BRAF inhibitors does not stop cells from proliferating, as they do not require BRAF in order to grow.

### MAP3K8 overexpression

Overexpression of MAP3K8, a gene that encodes the COT protein, has also been associated with resistance to BRAF inhibitors. A study noted that depleting V600E BRAF levels in cells correlated with increased levels of COT [32]. Notably, COT could independently activate the MAPK/ERK pathway [32]. Thus, patients with intrinsic overexpression of this protein, who are exposed to BRAF inhibitors, respond by producing further excessive amounts of COT and, rather than slowing cellular proliferation, the tumor burden increases.

### Hepatocyte growth factor (HGF) secretion by stromal cells

Secretion of HGF by stromal cells leads to activation of MET, a receptor for HGF. Binding of HGF to MET could reactivate the MAPK/ERK and PI3K/AKT pathways and therefore lead resistance to BRAF inhibition [33]. The identification of this mechanism was interesting as it demonstrated that the microenvironment of the tumor could influence its resistance to therapy.

### Acquired resistance to BRAF inhibitors

Patients who initially respond to BRAF inhibitor treatment, most often eventually acquire resistance. The most common mechanism of acquired resistance is via reactivation of the MAPK/ERK pathway, which can occur upstream, downstream or at the level of BRAF. Mechanisms of acquired resistance include (summary in Table 2):

### Upstream reactivation of MAPK/ERK

Upstream reactivation is thought to occur via upregulation of receptor tyrosine kinases (Figure 1A), resulting in continued cellular proliferation via ARAF and CRAF kinases, instead of BRAF kinase. BRAF^V600E^ melanoma treated with BRAF inhibitors may acquire drug resistance through flexible switching between different RAF isoforms capable of reactivating the ERK pathway. Three main RAF isoforms exist: ARAF, BRAF and CRAF [23]. Cancer cells may switch between these RAF variants, upregulating ARAF or CRAF when BRAF is blocked [34]. One mechanism is ERK negative feedback on RAS: Treatment with BRAF inhibitors arrests tumor growth by inhibiting the ERK pathway. Blockage of this pathway relieves ERK negative feedback on RAS, partially restoring RAS activity. RAS activation leads to RAS-induced BRAF^V600E^ dimers. BRAF inhibitors bind one component of each dimer and transactivate the other unbound molecule. This partially reactivates ERK signaling and reduces the long-term efficacy of BRAF inhibitor therapy [35].

The MAPK/ERK pathway can also be reactivated upstream of BRAF by mutations in RAS, meaning proliferation can continue, as RAS acts on ARAF and CRAF and compensates for the loss of BRAF [36]. These pathways could act as alternative survival routes for melanoma cells when BRAF signaling has been inhibited [37]. Mutated RAS-GTP cannot return to its inactive GDP-bound state and becomes constitutively active. Mutant RAS-GTP enhances BRAF^V600E^ dimerization, reactivates the ERK pathway and confers resistance to BRAF inhibitors since these drugs only block monomeric BRAF^V600E^ [38].

### Downstream reactivation of MAPK/ERK

This is thought to occur mainly through activating mutations in mitogen-activated protein kinases, MEK1/ MEK2. This may overcome BRAF inhibition, as MEK reactivation means that the need for BRAF to stimulate production of MEK is removed.

### Reactivation of MAPK/ERK at the level of BRAF

Reactivation at the level of BRAF itself can occur in several ways, many of which result in amplification of the mutant BRAF allele [39]. In many cases, copy number amplification means BRAF is overexpressed. Consequently, normal doses of BRAF inhibitors cannot sufficiently inhibit the increased levels of BRAF. Increased BRAF^V600E^ levels due to gene copy number gain may also promote spontaneous BRAF^V600E^ dimerization, reactivating the ERK pathway and causing treatment failure in some patients [39]. This has been described as an example of ‘drug-saturable’ resistance since administering higher doses of vemurafenib, to cell lines in which BRAF was over-expressed, demonstrated that the cells were still sensitive to the drug: it was simply that the BRAF inhibitor was impeded by the increased BRAF levels [39]. Furthermore, a BRAF^V600E^ splice variant called p61BRAF^V600E^ was detected in a subset of patients with acquired resistance to vemurafenib [40]. The variant could form dimers in a RAS-independent manner, making the BRAF inhibitor ineffective since it can only block monomeric BRAF^V600E^. Generation of such isoforms is probably due to mutations or epigenetic changes that affect BRAF splicing [41].

### Upregulation of PI3K/AKT

The PI3K/AKT-pathway interacts with several components of the ERK-pathway and the inhibition of either pathway may upregulate the other. Blockage of ERK signaling may lead to adaptive PI3K/AKT hyperactivity that compensates for BRAF inhibition and drives resistance [42]. Abnormal PI3K/AKT-signaling is a common feature of melanomas and causes resistance by stimulating alternative pathways that decrease dependence on ERK signaling. Mutations leading to upregulation of the PI3K/AKT pathway have been identified in 22% of melanomas with acquired resistance to BRAF inhibition [43]. A study found that within days of administering BRAF inhibitors, subsequent increased levels of AKT occur [44]. The study hypothesized that there would be a strong selective pressure towards cells with gain-of-function mutations, leading to increased activity of the PI3K/AKT pathway, in the presence of MAPK pathway inhibition. These cells would proliferate as they would have a survival advantage by not being affected by BRAF inhibition, and may account for the presence of an even greater tumor burden in patients who have responded to BRAF inhibition but then develop resistance [44]. The PI3K/AKT-pathway is activated by growth factors that bind to RTKs, such as PDGFR-β and IGF-1R. When BRAF is blocked, tumor cells may increase PDGFR-β and IGF-1R expression, leading to persistent PI3K/AKT-signaling that prevents apoptosis and promotes survival [45]. High surface expression of these receptors is associated with acquired resistance to vemurafenib both *in vitro* and *in vivo* [34]. Furthermore, PI3K and AKT-activating mutations may enhance AKT signaling, which increases anti-apoptotic signals and upregulates expression of key proliferative genes. These changes allow cancer cells to survive and replicate independently of BRAF, giving rise to acquired resistance [46].

### Enhanced EGFR signaling in low SOX10-expressing melanomas

Upregulation and activation of the epidermal growth factor receptor (EGFR) may also be associated with resistance to BRAF or MEK inhibition. Suppression of SOX10 in a proportion of melanomas can lead to TGF-β signaling and consequently to EGFR and platelet-derived growth factor receptor-β (PDGFRB) upregulation, conferring oncogene-induced senescence. This is reversed with BRAF or MEK inhibition, allowing low-expressing SOX10 tumor cells to be enriched in response to treatment [47].

Key aims of future research regarding resistance to current therapies include: further elucidating mechanisms of resistance and determining their relative power in reducing treatment efficacy; determining if certain mechanisms develop as a result of particular therapies; discovery of further shared mechanisms of resistance between classes of therapy, which may advise choice of further therapy; and identifying biomarkers of the patient's intrinsic resistance state, before initiation of therapy, in order to predict response.

## EFFORTS TO IMPROVE RESPONSES THROUGH THERAPEUTIC COMBINATIONS

Many of the mechanisms by which BRAF resistance develops involve alternate ‘survival pathways’, through which melanoma cells circumvent the role of BRAF, and thus continue to proliferate. Therefore, it has been reasoned that by using multiple inhibitors concurrently these alternate survival pathways could be simultaneously targeted to prevent, or overcome, resistance and improve survival. These concepts have resulted in the approval of treatment combinations (Table 1), and have given rise to several clinical trials (Table 3).

**Table 3:** Current phase III trials of combination therapies in melanoma. Information sourced from ClinicalTrials.gov

**Combinations of pathway inhibitors**				
**ClinicalTrials.gov Identifier**	**Drug type**	**Treatment combinations**	**Indication and inclusion criteria**	**Status**
NCT01584648	BRAF inhibitor + MEK inhibitor	Comparing dabrafenib + trametinibvs.Dabrafenib monotherapy	Stage IIIC (unresectable) or Stage IV BRAF V600E/K-mutant melanomaFirst line treatment	Active, not recruiting
NCT01682083	BRAF inhibitor + MEK inhibitor	Dabrafenib + trametinib vs.placebo	Adjuvant treatment of high risk V600E/K mutation-positive melanoma after surgical resection	Active, not recruiting
NCT01909453	BRAF inhibitor + MEK inhibitor vs. BRAF inhibitor alone	LGX818 + MEK162vs.vemurafenib monotherapyorLGX818 monotherapy	Locally advanced, unresectable or metastatic BRAF V600E/K-mutant melanomaFirst line treatment or second line in patients who have progressed on or after first line immunotherapy	Active, not recruiting
NCT01597908	BRAF inhibitor + MEK inhibitor vs. BRAF inhibitor alone	Dabrafenib + trametenibvs.vemurafenib monotherapy	Stage IIIC (unresectable) or Stage IV BRAF V600E/K-mutant melanomaFirst line treatment	Active, not recruiting
NCT01689519	BRAF inhibitor + MEK inhibitor vs. BRAF inhibitor alone	vemurafenib + cobimetinibvs.vemurafenib monotherapy	Stage IIIC (unresectable) or Stage IV BRAF V600E/K-mutant melanomaFirst line treatment	Active, not recruiting
**Combination of pathway inhibitors with immunotherapies**				
ClinicalTrials.gov **Identifier**	**Drug type**	**Treatment combinations**	**Indication and inclusion criteria**	**Status**
NCT02908672	Anti-PDL1 antibody + MEK inhibitor + BRAF inhibitor vs. placebo + MEK inhibitor + BRAF inhibitor	Atezolizumab + cobimetinib + vemurafenibvs.placebo + cobimetinib + vemurafenib	Stage IIIC (unresectable) or Stage IV BRAF V600E/K-mutant melanomaFirst line treatment	Recruiting
NCT02967692	Anti-PD-1 antibody + BRAF inhibitor + MEK inhibitor vs. placebo + BRAF inhibitor + MEK inhibitor	PDR001 + dabrafenib + trametinibvs.placebo + dabrafenib + trametinib	Stage IIIC (unresectable) or Stage IV BRAF V600E/K-mutant melanomaFirst line treatment	Recruiting

### Combination therapies targeting the MAPK/ERK pathway

Since MEK activation has been identified as an important mechanism of BRAF inhibitor resistance, a potential synergistic effect of combining BRAF and MEK inhibitors has been sought. The combination of dabrafenib and trametinib has been evaluated in phase III trials [48, 49]. In one trial (COMBI-d), patients were randomised to either dabrafenib and placebo or dabrafenib and trametinib. The ORR was 54% *vs.* 76% and the median PFS 8.8 months (95% CI 5.9–9.3) vs. 11.0 (95% CI 8·0–13·9) months (HR 0·67, 95% CI 0·53–0·84; *p* = 0.0004) with monotherapy vs. combination treatment [48]. In another trial, dabrafenib and trametinib were compared to vemurafenib alone, showing ORR of 64% vs. 51% (*P* < 0.001) and median PFS 11.4 months vs. 7.3 months (HR 0.56; 95% CI, 0.46–0.69; *P* < 0.001) [49]. The merits of combination therapies were also supported by findings from the coBRIM study, that compared vemurafenib combined with the MEK inhibitor cobimetinib vs. vemurafenib alone in previously untreated unresectable BRAF-mutant melanoma. Combination therapy demonstrated superior efficacy to monotherapy as the ORR and PFS were again significantly higher in the combination arm: ORR for combination therapy was 68% compared with 45% in the control group (*P* < 0.001); median PFS was 9.9 months in the combination group vs. 6.2 months in the control group (HR 0.51; 95% CI 0.39–0.68; *P* < 0.001) [50]. Administering combinations of BRAF and MEK inhibitor drugs may thus slow the development of resistance, as tumors cannot use the alternate MEK survival pathway to proliferate.

Furthermore, the recent development of potent highly selective BRAF and MEK inhibitors with unique pharmacologic profiles, has led to the potential for improved survival of patients with locally advanced or metastatic melanoma. The COLOMBUS trial compared the combination of the selective BRAF inhibitor encorafenib and the selective MEK inhibitor binimetinib versus treatment with vemurafenib or encorafenib alone. A median PFS of 14.9 months for the combination therapy compared to 7.3 months for vemurafenib (HR 0.54; 95% CI 0.41–0.71; *P* < 0.001) was reported in patients with BRAF mutant melanoma, with a favorable safety profile for the combination [51]. While the combination of encorafenib and binimetinib did not significantly improve survival compared with encorafenib alone (14.9 months vs. 9.6 months, HR 0.75; 95% CI 0.56–1.00, *P* = 0.051), these results still emphasize the potential benefit of using highly-selective BRAF and MEK inhibitors alone or in combination [51].

Another study however, found that administration of BRAF inhibitors not only led to acquired resistance to BRAF inhibitors, but also conferred resistance to MEK inhibitors, suggesting that combination therapy slows down but does not prevent the development of resistance [52]. It is worth noting that this study was carried out on cell lines, rather than *in vivo*, however the findings identified that BRAF-resistant cell clones were less sensitive to MEK inhibition as well as combined BRAF/MEK inhibition compared with parental cells, suggesting shared mechanisms of resistance [52]. This may suggest that additional inhibition of *different* signaling pathways outside of MAPK/ERK, may better overcome BRAF resistance (Figure 1A).

Recently, the potential of intermittent dosing schedules of BRAF and MEK inhibitors to extend disease control in BRAF mutant melanoma has been explored. In an *in vivo* xenograft model of melanoma, intermittent dosing of vemurafenib was shown to delay the onset of drug resistance compared to continuous dosing. The authors suggested that intermittent dosing significantly delayed the onset of drug resistance by exploiting the ‘fitness deficit’ shown by drug-resistant tumour cells in the absence of drug, potentially by reversing epigenetic mechanisms [53]. In concordance, a recent clinical case report described ongoing complete remission in a patient treated with an intermittent dosing regimen of vemurafenib followed by cessation of vemurafenib therapy [54]. Similarly, a recent retrospective study which examined re-challenging patients previously treated with a BRAF inhibitor, with a BRAF +/− MEK inhibitor following subsequent treatment or a treatment break, demonstrated clinically significant responses to re-challenge [55]. Intermittent dosing of BRAF/MEK inhibitor combinations has also been suggested to delay the emergence of resistance in BRAF mutant melanoma, and this is currently being investigated in a randomized phase 2 clinical trial of continuous versus intermittent dosing of dabrafenib with trametinib [56].

While beyond the scope of this review, it is noteworthy that ERK inhibitors including ulixertinib have undergone promising phase I trials in patients with V600E-positive and negative melanoma, pointing an additional future treatment avenue [57].

### Combining immunotherapy and BRAF inhibition

#### The promise of immunotherapy for melanoma

Immunotherapy involves utilizing the body's immune system to target cancer cells. The emergence of immunotherapies, especially checkpoint inhibitor antibodies, has provided the impetus for considering these in combination therapies with pathway inhibitors (Table 3).

CTLA-4 (Cytotoxic T-lymphocyte-associated protein 4) is a checkpoint molecule expressed on the surface of T cells that is involved in the suppression of T cell activity through competing with the co-stimulatory CD28 molecule for binding to CD80 and CD86, on the surface of antigen-presenting cells [58]. The monoclonal antibody ipilimumab is designed to target CTLA-4 and interfere with its T cell blocking functions, thus increasing the activation state and responsiveness of T cells. Ipilimumab was licensed by the Food and Drug Administration (FDA) for the treatment of malignant melanoma in 2011. This approval followed a successful phase III trial in which ipilimumab improved overall survival in unresectable stage III or IV disease in patients who had progressed on prior therapy [59]. Patients were randomized to ipilimumab, ipilimumab plus glycoprotein 100 peptide vaccine (gp100), or gp100 alone. Median overall survival in the ipilimumab and gp100 combination group was 10.0 months (95% CI, 8.5–11.5), in the ipilimumab alone group was 10.1 months (95% CI, 8.0–13.8) as compared with 6.4 months (95% CI, 5.5–8.7) in the gp100-alone group (HR 0.68; *P* < 0.001 and 0.66; *P* = 0.003 respectively) [59]. This study identified ipilimumab as the first agent to show a survival benefit in melanoma for decades.

Similarly, another checkpoint molecule, the programmed cell death protein 1 (PD-1), is expressed by B and T cells, and is upregulated by antigen-educated lymphocytes as a means of switching off cell activation and limiting normal tissue damage [60]. Melanoma cells express the PD-1 ligand (PD-L1), which can recognize PD-1 on T cells and can suppress T cell activity. Pembrolizumab and nivolumab are anti-PD-1 antibodies thought to act by preventing PD-L1 engagement of PD-1 on the surface of T cells, thereby preventing T cell anergy or deletion. In late stage clinical trials, these agents showed ORR of 30–40% and were approved as monotherapies by the FDA and the European Medicines Agency (EMA) in 2014.

In a pivotal phase III trial, pembrolizumab (10 mg/kg) every 2 or 3 weeks or ipilimumab (4 doses at 3 mg/kg) every 3 weeks [61]. ORR were 33.7% for pembrolizumab every 2 weeks and 32.9% for pembrolizumab every 3 weeks compared with 11.9% for ipilimumab. After a 7.9-month median follow up, ongoing responses were recorded at 89.4% for pembrolizumab every 2 weeks, 96.7% for pembrolizumab every 3 weeks, and 87.9% for ipilimumab. The 12-month survival rates were 74.1%, 68.4% and 58.2%, respectively (pembrolizumab every 2 weeks, HR 0.63; 95% CI, 0.47–0.83; *P* < 0.0005, and pembrolizumab every 3 weeks, HR 0.69; 95% CI, 0.52–0.90; *P* = 0.0036) each compared with ipilumumab [61]. These findings led to the regulatory approval for pembrolizumab (dosed every three weeks) for the treatment of melanoma by the FDA and the EMA.

In the Checkmate 067 phase III study, nivolumab plus ipilimumab combination treatment was compared with nivolumab or ipilimumab monotherapies in patients with metastatic melanoma. Nivolumab and ipilimumab combination treatment resulted in PFS of 11.5 months (HR 0.42, 99.5% CI, 0.31–0.57; *P* < 0.001); 6.9 months for nivolumab (95% CI, 4.3–9.5) (HR 0.57; 99.5% CI, 0.43–0.76; *P* < 0.001) and 2.9 months or ipilimumab (95% CI, 2.8–3.4) [62]. Nivolumab plus ipilimumab combination treatment was approved by the FDA in 2015. A recent update on CheckMate 067 showed a superior ORR for ipilmumab plus nivolumab of 58.9 months (95% CI 53.3–64.4) compared to 44.6 (95% CI 39.1–50.3) for nivolumab and 19.0 months (14.9–23.8) for ipilimumab [63]. Notably the emergence of immune-related adverse events (irAEs) with the checkpoint inhibitors vary in frequency and intensity depending on the target and regimen used [64]. The CTLA-4-targeting antibodies confer greater toxicity than antibodies targeting the PD-1/PD-L1 axis. Combining CTLA-4 and PD-1 therapy leads to a sharp rise in the risk of severe toxicity (requiring hospitalization in over a third of patients treated on the Checkmate 067 protocol) [62].

Since only a proportion of patients benefit from checkpoint inhibitor therapy, and in light of often significant adverse events observed in the clinic, there remains a need for standardized patient stratification in order to determine which patients are likely to benefit. An example of patient stratification is seen in non-small cell lung cancer (NSCLC), where PD-L1 tumor status is used as a biomarker of potential response to pembrolizumab therapy [65]. Despite benefits to be had from identifying patients who will respond to anti-PD-L1 antibodies, PD-L1 status is not completely reliable for stratification in melanoma. Chemotherapy has been suggested to alter PD-L1 expression levels so that status may change following therapy, and a standardized cut-off point for what constitutes a ‘PD-L1 negative’ tumor has not yet been established [66]. More research is therefore required so that reliable biomarker testing can be implemented, to improve selection for checkpoint inhibitor treatment for patients with melanoma.

#### Molecular and immunological resistance to immunotherapy

Despite encouraging results, as with BRAF/MEK inhibitors, immunotherapy too encounters problems in the form of resistance. The mechanisms of resistance to immunotherapy continue to form the subject of intense study. Of interest, the effect of copy number loss of tumor suppressor genes, such as PTEN has been implicated in disease progression (Figure 1B). In 2017, a study of integrated molecular analysis of tumor biopsies, demonstrated that high burden of copy number loss was associated with disease progression despite sequential CTLA-4 blockade, and PD-1 blockade, compared with those biopsies from patients who responded to CTLA-4 blockade [67]. The authors also demonstrated that T cell receptor (TCR) clonality positively correlated with both treatment response and with pre-treatment immune scores (used to estimate immune activation, within the tumor microenvironment), with regards to PD-1, but not to CTLA-4, blockade [67]. Loss of PTEN on chromosome 10 increased the odds of resistance 5.58-fold, compared with patient tumors with no PTEN loss [67].

Studies also point to tumor-associated macrophages as potential decoy cells by sequestering anti-PD-1 antibodies away from their T cell targets through Fc gamma receptor engagement [68]. Such mechanisms suggest that perhaps reducing the macrophage and immunomodulatory cell infiltrate in tumors by pathway blocking agents may be beneficial prior to or alongside checkpoint therapy. Alternatively, novel antibody engineering approaches including enhancing target engagement and improving antibody activatory effector functions or strategies that might be able to activate tumor-associated macrophages and other effector cells against tumors may help overcome resistance [69].

#### Rationale and clinical study of pathway inhibitor combinations with immunotherapy

While checkpoint blocking antibodies have been independently effective at increasing survival of subsets of patients with melanoma, combining immunotherapies with BRAF and MEK inhibitors is an enticing prospect. Identifying the right combination of immunotherapy and BRAF and/or MEK inhibition may confer improved and more sustained responses, even in the face of BRAF inhibitor resistance [70].

From a clinical viewpoint, MAPK pathway inhibitors induce a relatively short-lived tumor response in most patients with BRAF-mutated melanoma. Furthermore, most patients still do not gain long term benefit from checkpoint inhibitors. For those patients who respond, disease control can be maintained for many years. Therefore, it is plausible that pathway inhibitor/immunotherapy combinations may induce maintained responses in a greater proportion of patients.

From a biological perspective, it is hypothesized that MAPK pathway inhibitors may potentiate an anti-tumor immune response, which could then be enhanced through immune checkpoint inhibition. Additionally, destruction of cancer cells by MAPK pathway inhibitors could reduce the immunosuppressive effects conferred by tumor environments. This may allow immunotherapeutic agents to act more effectively in favor of T cell activation and survival of tumor antigen-specific T cell clones. Studies from pre-clinical models of melanoma and those from patients treated with vemurafenib and dabrafenib report evidence of enhanced infiltrating T cell levels, reduced CD11b+/Gr-1+ MDSC and reduced FoxP3+ Treg frequencies in tumors [71]. These studies may suggest that immunological activation may associate with and complement responses to MAPK pathway inhibitors [72].

Furthermore, tumor cell death effected by pathway inhibitors may also lead to increased tumor antigens available for uptake by antigen-presenting cells and presentation to cognate T cells [73]. When combined with T cell-activating immunotherapies, BRAF inhibitors, and possibly combinations of BRAF and MEK inhibitors, may augment adaptive immune responses against cancer cells. This notion is supported by pre-clinical findings that BRAF inhibitor therapy can enhance IFNγ production, T cell proliferation and MHC expression by melanoma cells, and by clinical evidence of increased tumor-infiltrating lymphocyte populations during BRAF inhibitor therapy [71, 74–75]. In further support, BRAF^V600E^ inhibitor treatments have been associated with dendritic cell maturation, increased expression of co-stimulatory molecules including CD40L, production of IFNγ, TNFα and IL-12, and moderate enhancement of circulating tumor antigen-reactive CD8+ T cells [76, 77–78]. Pre-clinical evidence of combinations of BRAF inhibition, MEK inhibition and anti-PD-1 antibody treatment also suggest superior anti-tumor effects compared with monotherapy [79]. This was corroborated by experimental findings of enhanced effects with BRAF and MEK inhibitor combinations together with either adoptive cell transfer or with anti-PD-1 therapy, associated with heightened immune marker expression, MHC upregulation and T cell infiltration [80, 81]. Immunological mechanisms may also be responsible for early reports of clinical benefits from combination therapy with the anti-PD-L1 antibody atezolizumab and the MEK inhibitor cobimetinib in patients with BRAF^V600E^ mutant and BRAF wild-type melanoma. It has been postulated that the pathway inhibitor may sensitize tumors by supporting enhanced MHC Class I expression and infiltration of cytotoxic T cells A planned phase III trial will compare the combination of a PD-L1 inhibitor with a MEK inhibitor versus a PD-L1 inhibitor alone.

In the same way that melanoma has been postulated to share resistance mechanisms to both BRAF and MEK inhibitors, a major limitation to the success of future combination therapies, could be conferred resistance to BRAF/MEK inhibitors and immunotherapy. As previously mentioned, PTEN loss has been strongly implicated as a mechanism to intrinsic BRAF resistance. Hence loss of this tumor suppressor gene, via mutations, methylation or high copy number loss, may represent a shared mechanism of resistance between BRAF inhibitors and immunotherapy (Figure 1B).

There are currently a few clinical trials underway aimed at assessing potential clinical applications of different pathway inhibitor treatment combinations with immunotherapies (Table 3). However, results to-date suggest that combination therapy may be hampered by significant toxicities such as hepatotoxicity and bowel perforation, and this may undermine efficacy in the long term [82, 83]. Data from a phase I study of the combination of BRAF/MEK inhibition and an anti-PD-1 antibody demonstrated increased levels of tumor-infiltrating T cells even post-treatment, a potential indication of increased immune activation [84]. However, ongoing overall response rates to this triple therapy do not appear to be better than the response rate to BRAF and MEK inhibition dual therapy [85]. Further understanding of the mechanisms of resistance to BRAF/MEK inhibitors and immunotherapy is required to determine if conferred resistance between all of these classes, could be responsible for current response rates. Nevertheless, further studies, examining dual treatment combinations of MAPK pathway and immune checkpoint inhibitors, provide hope that some combined treatment regimens may be better tolerated [86].

## SEQUENCING OF THERAPY, GENOMIC CORRELATES AND THE POTENTIAL OF PATIENT STRATIFICATION

There has been a relatively quick transition from having few therapeutic options for the treatment of melanoma before 2011, to FDA and EMA approval of pathway inhibitors and of checkpoint blocking antibodies. To-date, the EMA and the FDA have approved vemurafenib, dabrafenib, and trametinib as monotherapies, combined dabrafenib and trametinib, and combination of vemurafenib and cobimetinib; ipilimumab, nivolumab, pembrolizumab and T-Vec monotherapies, and combined ipilimumab and nivolumab (Table 1). The next challenge is to discern the optimum order in which to administer agents, in order to gain the maximum clinical benefits for melanoma patients.

It has been postulated that the right sequence of treatment may hold the key to improving responses and survival [87]. For instance, first line BRAF/MEK inhibition followed by an immune checkpoint inhibitor, may allow a quick initial response to treatment and the ability to prime the immune system before administering immunotherapy. On the other hand, first line immunotherapy may provide an opportunity to benefit patients by triggering immunological memory and the possibility of discontinuing treatment whilst maintaining a response – not an option with BRAF inhibition. It is hoped that future trials will ascertain an optimal treatment pathway.

Mechanisms of immune evasion in melanoma are plentiful. Mutant BRAF not only promotes melanocyte proliferation but has also been shown to manipulate the tumor microenvironment by increasing the release of IL-6, IL-10 and VEGF [73]. By increasing levels of these immunosuppressive mediators and recruiting regulatory T cells BRAF mutations may promote immune tolerance, conferring a survival advantage to malignant cells [73]. On the other hand, recognition of mutant BRAF can induce an immune response. Synthetic BRAF^V600E^ has been shown to generate peptide-specific MHC class II-restricted CD4+ T cells, *in vitro* [88]. Another *in vitro* study demonstrated cytotoxic T cell activity, against melanoma cells with BRAF^V600E^ and HLA-A2 binding sites [89]. Whilst the mechanisms by which mutant BRAF induces immunogenicity are eclipsed by its varied instruments of immune evasion, V600E mutations could provide a known therapeutic target.

A 2015 study using next generation sequencing in 10 patients with BRAF^V600E^ found that 2 out of 3 patients, with long term complete response, had no other mutation in BRAF or other genes [90] However, the third patient not only had another BRAF mutation (T5995), but an aurora kinase a amplification [90]. The relative importance of non-V600E mutations (including other BRAF mutations and those in other genes) in predicting disease progression, response to and possible mechanisms of resistance to therapeutic interventions, is unknown.

Mutational load, neoantigen load and cytolytic activity within the immune microenvironment correlate significantly with clinical benefit in patients with metastatic disease receiving ipilimumab [91]. However, no specific sequences of recurrent neoantigens were found consistently in those with response to treatment [91]. This is supported by a 2016 study, which used an algorithmically-predicted mutational load, based on mutational status from cancer related genes in melanoma and lung cancer [92]. Predicted total mutational load (PTML) correlated with actual total mutational load as validated by whole exome sequencing datasets. PTML also positively correlated with clinical benefit and overall survival in melanoma patients receiving ipilimumab [92]. Another 2016 study examined the relationship of mutational load to PD-1 inhibitors in metastatic melanoma [93]. Mutational load was found to positively correlate with improved survival but, once again, no predictive recurrent neoantigens associated with response to PD-1 inhibitors were identified [93]. In further support of this, a previously mentioned 2017 study which found association between high burden of copy number loss and disease progression also analyzed the effect of mutational load [67]. Clinical benefit was associated with raised mutational load and lower copy number loss, the effects of which were found to be non-redundant [67]. Whilst mutational load was not found to differ between pre-treatment samples of responders or non-responders to either checkpoint inhibitor the authors postulated that this might be due to sample size [67].

Whilst mutational load has been shown to correlate with survival and response to treatment, patients with high mutational loads do not always respond to checkpoint inhibitors and some patients with low mutational loads have been shown to respond [93]. Therefore, at present, accurate prediction of survival and response cannot be made with mutational load alone.

Alternatively, the analysis of circulating cell-free tumor DNA (cfDNA) may provide a useful approach to assess prognosis, monitor therapeutic response and detect the onset of resistance [94]. In BRAF^V600E^ mutant melanoma, lower levels of BRAF^V600E^ cfDNA were associated with a higher ORR and increased PFS in patients treated with BRAF inhibitors [95]. Furthermore, cfDNA levels correlated with clinical and radiological outcomes in a group of patients receiving checkpoint inhibitor immunotherapy [96]. Gray et al. showed that plasma cfDNA reflected response to BRAF/MEK inhibitor treatment and could be used to predict the onset of acquired resistance in a subset of patients [94]. Following treatment initiation, plasma cfDNA concentrations declined to almost undetectable levels. Subsequently, cfDNA was elevated again in all cases of acquired resistance. In some patients, the increase in plasma cfDNA preceded detection of progressive disease by CT scans. Moreover, NRAS mutations conferring resistance to BRAF/MEK inhibitors were identified in the cfDNA of patients prior to radiological evidence of progression. Thus, circulating cfDNA could provide valuable prognostic information and may be used to track patient response and tumor evolution. Importantly, cfDNA analysis may help predict early resistance and allow switching to other more effective therapies [90]. Future studies in larger cohorts are needed to confirm the predictive value of cfDNA in melanoma.

Together, these observations mandate further dissection of the pathways involved in BRAF resistance. Defining reliable predicative markers of prolonged efficacy and intrinsic and acquired resistance to targeted therapy will enable accurate patient stratification and allow the implementation of highly personalized regimens.

## CONCLUSIONS

While resistance to BRAF inhibitors has limited their clinical benefits, the emergence of MEK and other pathway blockade drugs alongside a new generation of immuno-oncology agents, such as the checkpoint blocking anti-CTLA-4 and anti-PD-1 antibodies, have brought about the possibility of a range of treatment combinations. This prospect has raised hopes that better responses and prolonged survival for patients could be achieved. With some dual agent combinations already approved, promising pre-clinical findings of synergistic effects with triple or other combination treatments are yet to be translated into viable clinical treatments.

New insights may arise from the clinical experience gained with BRAF inhibitors. Activation of pathways such as the PI3K-Akt-mTOR could point to new targets and selection of treatment combinations that may help prevent or overcome resistance. The PI3K inhibitor buparlisinib was recently identified as being beneficial in melanoma brain metastases [97]. Other mechanisms such as those triggered by metabolic pathway alterations may also contribute to constitutive activation and, possibly, resistance [98]. The nature of immune responses before and during treatment and with the onset of resistance to pathway inhibitor drugs or combinations may also be of critical importance for selection of combinations with checkpoint blockade antibodies and with future immunotherapies [99, 100].

Also of the utmost importance to both patient comfort and safety would be to discover if certain combination therapies have different side effect profiles or predispose to serious adverse events. Combining multiple agents may narrow therapeutic windows, reducing quality of life. Benefit and safety results from current phase III triple therapy trials will need to be clearly beneficial for universal adoption.

With the treatment landscape of malignant melanoma now facing the real prospect of multi-drug combinations, numerous challenges remain. These include incomplete understanding of resistance mechanisms to these new therapies and their combinations, and lack of reliable biomarkers to select patients likely to benefit. The ultimate goal for the Multidisciplinary Team (MDT) is to be able to further personalize care, by offering tailored therapy, taking account of co-morbidities, genetics, treatment efficacy and understanding their patient's wishes and values. Alongside clinical testing and implementation, intense focus on dissecting mechanisms and biomarkers of treatment response may hold the key to these novel approaches.

## Abbreviations

AKT: Protein kinase B
ARAF: Serine/threonine-protein kinase A-Raf
BRAF: Serine/threonine-protein kinase B-Raf
CRAF: Serine/threonine-protein kinase C-Raf
CTLA-4: Cytotoxic T-lymphocyte-associated protein 4
ERK: Extracellular signal–regulated kinases
MAPK: Mitogen-activated protein kinase
MEK: Mitogen-activated protein kinase kinase
PD-1: Programmed cell death protein 1
PD-L1: Programmed cell death protein ligand 1
PI3K: Phosphatidylinositol-4,5-bisphosphate 3-kinase
PTEN: Phosphatase and tensin homolog
